# A Modified Roger’s Distance Algorithm for Mixed Quantitative–Qualitative Phenotypes to Establish a Core Collection for Taiwanese Vegetable Soybeans

**DOI:** 10.3389/fpls.2020.612106

**Published:** 2021-01-12

**Authors:** Chung-Feng Kao, Shan-Syue He, Chang-Sheng Wang, Zheng-Yuan Lai, Da-Gin Lin, Shu Chen

**Affiliations:** ^1^Department of Agronomy, College of Agriculture and Natural Resources, National Chung Hsing University, Taichung, Taiwan; ^2^Advanced Plant Biotechnology Center, National Chung Hsing University, Taichung, Taiwan; ^3^Biotechnology Division, Taiwan Agricultural Research Institute, Taichung, Taiwan; ^4^Plant Germplasm Division, Taiwan Agricultural Research Institute, Taichung, Taiwan

**Keywords:** vegetable soybean, edamame, germplasm, modified Roger’s distance, mixed quantitative-qualitative phenotypes, core collection, diversity, multiple imputation

## Abstract

Vegetable soybeans [*Glycine max* (L.) Merr.] have characteristics of larger seeds, less beany flavor, tender texture, and green-colored pods and seeds. Rich in nutrients, vegetable soybeans are conducive to preventing neurological disease. Due to the change of dietary habits and increasing health awareness, the demand for vegetable soybeans has increased. To conserve vegetable soybean germplasms in Taiwan, we built a core collection of vegetable soybeans, with minimum accessions, minimum redundancy, and maximum representation. Initially, a total of 213 vegetable soybean germplasms and 29 morphological traits were used to construct the core collection. After redundant accessions were removed, 200 accessions were retained as the entire collection, which was grouped into nine clusters. Here, we developed a modified Roger’s distance for mixed quantitative–qualitative phenotypes to select 30 accessions (denoted as the core collection) that had a maximum pairwise genetic distance. No significant differences were observed in all phenotypic traits (*p-*values > 0.05) between the entire and the core collections, except plant height. Compared to the entire collection, we found that most traits retained diversities, but seven traits were slightly lost (ranged from 2 to 9%) in the core collection. The core collection demonstrated a small percentage of significant mean difference (3.45%) and a large coincidence rate (97.70%), indicating representativeness of the entire collection. Furthermore, large values in variable rate (149.80%) and coverage (92.5%) were in line with high diversity retained in the core collection. The results suggested that phenotype-based core collection can retain diversity and genetic variability of vegetable soybeans, providing a basis for further research and breeding programs.

## Introduction

Soybean [*Glycine max* (L.) Merr.] is a legume crop that is rich in protein, oil, and other nutrients such as lecithin and isoflavones. It is one of the most important economic crops worldwide (Liu, 1997). Owing to the high nutritional value, soybeans are good plant-based protein foods. The soy protein is regarded as a complete protein because it contains essential amino acids in animal proteins (Velásquez and Bhathena, 2007). Mainly, soybeans are classified into grain soybeans and vegetable soybeans according to their harvest period. Briefly, grain soybeans harvested at reproductive stage 8 (R8) are processed as oil products, animal feeds, and soy products. On the other hand, vegetable soybeans, also known as edamame, that were harvested at reproductive stage 6 to 7 (R6–R7) are taken as snacks and vegetables (Fehr, 1971; Young et al., 2000; Reddy et al., 2019).

In contrast to grain soybeans, vegetable soybeans are characteristic of the green-colored pods and seeds, larger seeds (over 30 g/100 seeds), smooth texture, less beany flavor, and higher contents of vitamin A, vitamin C, sucrose, and starch (Saldivar et al., 2010; Zhang et al., 2013). The demand for vegetable soybeans has increased as a result of changes in eating habits nowadays. Vegetable soybeans are consumed as frozen edamame or vegetables in Taiwan, Japan, China, and South Korea (Konovsky et al., 1994; Hu et al., 2007). This kind of soybean tastes sweet because it was picked at a high sugar level, and it contains high calcium and approximately 13% of protein (Dwevedi and Kayastha, 2011). Due to the abundant phytochemicals in edamame, it is known as a functional food (Mebrahtu, 2008). In recent years, the awareness of food nutritive value and health benefit brings a steady increase in the demand and also the planted area of vegetable soybeans in the United States (Jiang et al., 2018). Therefore, vegetable soybeans are good nutritional crops with high commercial value.

Soybeans are rich in isoflavones and anthocyanins, which prevent or inhibit the progression of cancer and other chronic diseases (Magee et al., 2004; Messina, 2016; Ziaei and Halaby, 2017). Evidence showed that breast cancer incidence is much lower in Asians than other populations resulting from the intake of isoflavones in soybeans in their daily diet (Adlercreutz, 2002; Mense et al., 2008). Anthocyanins are neuroprotective agents in the central nervous system, participating in mechanisms of suppression of neuroinflammation and oxidative stress (Zafra-Stone et al., 2007; Zhang et al., 2019). High isoflavone and anthocyanin contents of soybeans are value-added nutrition, as well as soy products. It was pointed out that isoflavone and anthocyanin contents in soybeans can be influenced by genotypes, food processing, and cultivated environment (Kim et al., 2014; Miladinović et al., 2019). On top of that, anthocyanins are abundant in black soybean seed coat, and isoflavones are positively correlated to plant height, effective branches, number of pods per plant, and number of seeds per plant (Choung et al., 2001; Zhang et al., 2014). Our vegetable soybean germplasms provide detailed information of those phenotypic traits, which could be quality data for isoflavones and anthocyanins, for establishing a core collection (CC).

Apart from the amount of nutrient composition in vegetable soybeans, the quality of vegetable soybeans depends on the external appearance (Shanmugasundaram, 1991). To market, a good-quality vegetable soybean consists of large seeds, bright green pods, light color pubescence (white to gray), and two seeds per pod at least (Johnson et al., 1999). The pod color is affected by genotype, planting density, and processing procedure (Zeipiòa et al., 2017). Seed size is a measure of seed length, width, and thickness (Xu et al., 2011). One hundred-seed weight, affected by seed size, is significantly correlated with pod length and pod width (Xu et al., 2011). Besides, a narrow leaflet is related to a greater number of three- and four-seeded pods (Bravo et al., 1980; Frank and Fehr, 1981; Shanmugasundaram, 1991). Additionally, soybean is a chilling-sensitive crop, and it reduces the growth potential when the temperature is below 20°C (Hofstra, 1972). The chilling temperatures (about 15°C) are an unfavorable condition that would affect the growth and the development of soybeans, in particular, the early stage of the growth. In the flowering stage, it may cause flower abscission and pod setting failure. In the pod and grain filling stage, grains would fill poorly (Kurosaki and Yumoto, 2003). Low temperatures result in the reduction of total seed weight per plant and of the pod number per plant in soybeans (Ikeda et al., 2009). Low temperatures in the flowering stage cause browning and cracking of the seed coat in soybeans, leading to lower market value due to poor appearance quality (Sunada and Ito, 1982; Githiri et al., 2007). In the past, during 1960–1985, farmers in Taiwan grew grain soybeans mostly, and breeders under the Council of Agriculture (COA) conducted crossbreeding programs resulting in several soybean varieties; therefore, most of our previous germplasms were for grain soybeans. However, the cheap imported soybean replaced most of the Taiwanese self-production and provided the most requirement of the soybean industry in Taiwan. Since the 1980s, our soybean production has turned to the goal of producing high-quality vegetable soybeans, and that is the reason why few collections mainly aimed for breeding vegetable soybean varieties by the 1990s. A large-scale soybean germplasm collection and renewal was started in 1986 led by National Chung-Hsing University with soybean breeders from other institutions, including the Asian Vegetable Research and Development Center (AVRDC), the Taiwan Agricultural Research Institute (TARI), National Chiayi University, and many local agricultural experiment stations under COA. The collections were from around Taiwan island including wild species and introducing those from our technological teams around the world. These results enriched our germplasm collection in various ways, genetic variation and quantity included. Additionally, for some of them, their origin can be recognized using the accession number (Lin, 1997). To make it usable for practical plant breeding, we decided to generate a CC from germplasms of vegetable soybean.

The concept of a CC, defined as a limited set of accessions, was first introduced by Frankel (1984). The CC is representative of the whole accessions, with minimum repetitiveness and maximum diversity. There are many studies on CC for soybean (Wang et al., 2008; Qiu et al., 2013; Guo et al., 2014), but no CC for vegetable soybean. This is because vegetable soybean accessions are quite hard to acquire. The major reason is that vegetable soybean is harvested at an immature stage and thus requires specific planting to collect seeds for a special purpose. In particular, after vegetable soybean is harvested, there will be no grain soybeans. Also, a small consumer market and less attention are other reasons. To date, there are only a few studies on vegetable soybean germplasm (Jiang et al., 2018). The first genetic diversity investigation of vegetable soybean was by Mimura et al. (2007). They used 122 alleles detected by 17 simple sequence repeats (SSRs) to investigate the genetic diversity among 130 accessions (107 from Japan, 10 from China, and 13 from the United States) of vegetable soybeans and found that Japanese edamame have a narrow diversity. Later, Zhang et al. (2013) used 22 expressed sequence tag-derived SSRs selected from grain soybean to access the genetic diversity of 48 vegetable soybean accessions (43 from China, 3 from Japan, and 2 from the United States) and found a narrow genetic base in Chinese vegetable soybean accessions (Zhang et al., 2013). Both studies only calculated genetic diversity of vegetable soybean accessions without establishing a CC. A possible reason may be due to the small collection of accessions and a narrow genetic base. A narrow genetic base may narrow variability coverage and further limit vegetable soybean breeding and variety improvement, as vegetable soybean requires a broad and diverse collection of accessions to produce high-quality products for the global market. To enrich the genetic diversity, it is necessary to introduce more foreign soybean varieties. With the nutritional value and benefit for neurological health in vegetable soybeans, it is vital for us to raise attention to the preservation of vegetable soybean resources.

The establishment of a CC is based on similarity among accessions. Similarity is generally defined as a distance-based measure in a multidimensional space. The similarity measures for quantitative traits are computed by using *Euclidean* distance and *Manhattan* distance, which are well-defined (Aggarwal et al., 2001). However, it is not straightforward for qualitative traits. To do this, a frequent-based approach (Rogers, 1972) can be applied for the purpose of calculating genetic distance among accessions for qualitative traits. A larger distance between two accessions represents dissimilarity between two accessions. It is important to extract information, for given accessions, accurately from phenotypic traits using correct similarity metrics, so that the interpretation of findings from trait-based studies is on a theoretically sound basis.

*k*-means has long been used for clustering. However, there is some weakness for *k*-means. First, the original version of *k*-means had the difficulty to handle qualitative data. Different initial seeds may lead to distinct results. It is also difficult to determine the number of clusters due to its nature of a supervised algorithm (Sajidha et al., 2020). By applying the dummy variable for qualitative traits, *k*-means can be modified as weighted *k*-means clustering (Huang et al., 2005; Foss and Markatou, 2018), so that it is able to deal with qualitative and quantitative traits at the same time. In addition, the weight of the noise can be reduced by determining the weight of qualitative and quantitative traits. The present study used weighted *k*-means to perform clustering for individual phenotype and for the overall phenotypes, in a base of unsupervised data learning, to determine a proper number of clusters automatically by the data learning.

Missing data in phenotypes are often seen in many germplasms due to problems with cultivation or negligence in investigation (Singh et al., 2019). Deletion of accessions with missing phenotypes and the utilization of simple imputation methods (e.g., mean or median substitution, regression imputation) should not be used to calculate trait-based diversity, because both methods do not take into account differences of functional plant traits between accessions (Taugourdeau et al., 2014). Multiple imputation estimated missing phenotypes by chained equations and produced a lower error and bias on the estimation of missing phenotypes as uncertainty is taken into account during computational process (Penone et al., 2014). In particular, it is superior and robust to deal with no more than 30% threshold of missing phenotypes (Taugourdeau et al., 2014). Multiple imputation requires high performance and a parallel computing system, especially for categorical variables.

In the present study, we proposed a modified Roger’s distance algorithm for mixed quantitative–qualitative phenotypes to establish a CC for breeders using 213 Taiwanese vegetable soybean germplasms. To downsize germplasms without losing much diversity (Frankel and Arber, 1984), we first constructed multiple imputation to predict missing phenotypes for constructing a completed (i.e., observed plus imputed values) dataset of phenotypes (i.e., observed plus imputed values), so that the weighted *k*-means clustering can be applied to search for the patterns and the genetic structure of germplasms in vegetable soybean. This provides an opportunity for a better understanding of genetic diversity among complex characteristics in accessions (Yun et al., 2015). We then performed a modified Roger’s distance algorithm using observed phenotypes of vegetable soybean germplasms to calculate pairwise genetic distance separately for quantitative and qualitative phenotypes among accessions to construct the CC of Taiwanese vegetable soybeans, followed by diversity calculation of individual phenotype and CC assessment.

## Materials and Methods

### Vegetable Soybean Germplasm

The germplasm of vegetable soybean composed of 213 accessions used in the present study was collected from the National Plant Genetic Resources Center (NPGRC) in Taiwan. The countries of origin of the accessions were grouped into Taiwan, Japan, United States, China, Hong Kong, and Philippines. Phenotypic traits were investigated and recorded in the field at Kaohsiung District Agricultural Research and Extension Station, COA, and followed the guidelines of distinctness, uniformity, and stability (DUS) test for vegetable soybean in four consecutive seasons (1995–1998 autumn). Each accession was characterized for 47 phenotypic traits in relation to morphology (38 traits), growth (5 traits), phenology (2 traits), and production (2 traits). For detailed agronomic descriptors, please refer to Supplementary Table 1. The phenotype data were in stacked format that cannot be directly used for statistical analysis. We first unstacked the phenotype data and found that no accessions have missing values for all 47 phenotypic traits. Thirteen pairs of accessions were identically redundant within and among all the phenotypic traits; hence, 13 accessions were randomly selected and excluded from each pair of accessions. In the present study, a total of 200 accessions with 29 phenotypic traits were retained for analysis, based on a threshold of slight to mild missing rate (<30%) in phenotype. There are 15 quantitative traits [seed length (mm), seed width (mm), seed thickness (mm), 100 seed weight (g), plant height (cm), leaflet length (cm), leaflet width (cm), pod length (cm), pod width (cm), stem length to first pod (cm), shelling rate (%), immature seed length (mm), immature seed width (mm), immature seed thickness (mm), and 100 immature seed weight (g)] and 14 qualitative traits (seed shape, seed coat color, hilum color, hypocotyl coloration, stem color, number of branches, leaflet size, leaflet shape, leaf color, plant type, pubescence density, pubescence color, corolla color, and pod set capacity). We denoted the 200 germplasm accessions as the entire collection (EC), where 29 phenotypic traits were used to construct a CC.

### Meteorology Data

The meteorology data, including temperature (°C), humidity (%), duration of sunshine (hours), precipitation (mm), and days with precipitation (days), were collected daily during 1995–1998 from the Kaohsiung District Agricultural Research and Extension Station and the Kaohsiung Weather Station of the Central Weather Bureau in Taiwan. One-way ANOVA was conducted to test for differences among four autumn seasons (October–November) and among 4 years, followed by paired samples *t*-test only when ANOVA *p*-value reached significant difference. The results suggested that no significantly environmental effects were observed on the phenotypes investigated in the field at Kaohsiung District Agricultural Research and Extension Station (Supplementary Table 2).

### Multiple Imputation of Incomplete Phenotypes

Multiple imputation is a mathematically computational method to deal with missing values in phenotypes (Lee and Simpson, 2014). There were three steps, consisting of imputation, estimation, and pooling, in multiple imputation. In the imputation step, missing values of a phenotypic trait were imputed by using Bayesian linear regression based on maximum completed data from the remaining traits to generate a completed dataset (i.e., observed plus imputed values). We repeated this procedure several times (say 30 times) to create multiple completed datasets in order to capture the uncertainty during the procedure. The model treated all variables (i.e., traits) as fixed and all error terms as random to maintain the natural variability in the data. In the estimation step, standard statistical analysis was performed for each of the completed datasets. In the pooling step, we aggregated the results derived from each of the completed data analyses. The three steps guarantee a more accurate and reliable data to obtain valid conclusions for downstream analysis (Royston, 2004). The mi package in R was used in the analysis. The values of qualitative traits were estimated from posterior predictive distribution, while quantitative traits were from predictive mean matching (Su et al., 2011; Kropko et al., 2017). Compared to other packages, Bayesian linear regression was applied in the mi package, which sorts out the issue of separation for qualitative traits. It can examine the collinearity in the data automatically. Four independent chains (i.e., chained equations) were used to account for the uncertainty between and within the completed datasets during missing data imputation. An $\hat{\text{R}}$ statistic was calculated to evaluate the iterative convergence (default is smaller than 1.1) of the multiple imputation (Su et al., 2011).

### Weighted *k*-Means Clustering

To examine the diversity of vegetable soybean germplasms, weighted *k*-means approach by kamila package in R (Lloyd, 1982; Huang et al., 2005; Foss et al., 2016; Foss and Markatou, 2018) was used to explore the feature of the germplasms. The weighted *k*-means clustering can handle both qualitative and quantitative data. Let *X* = {*X*_1_,*X*_2_,…,*X*_*n*_} be a set of *n* vegetable soybean accessions. Assume that each accession has *m* phenotypes. The objective function *P*(*A*,*W*,*C*) is calculated as

$$P\,\left(A,W,C\right)=\,\sum_{l=1}^{k}\sum_{i=1}^{n}\sum_{j=1}^{m}a_{i\,l}\,w_{j}\,d\,\left(x_{i\,j},c_{l\,j}\right)$$

where *a*_*il*_ is an *n*×*k* assignment matrix, which subjects to

$$a_{i\,l}\in \left\{0,1\right\},1\leq i\leq n,1\leq l\leq k$$

$$\sum_{l=1}^{k}a_{i\,l}=1,\forall i$$

*a*_*il*_ = 1⇔*x*_*i*_ belongs to the *l*th cluster

*w*_*j*_ is the weight parameter, which is defined by

$$w_{j}=\{\begin{array}{c} w,\mathrm{if}\,j\in \mathrm{qualitative}\,\mathrm{trait}\\ 1-w,\mathrm{if}\,j\in \mathrm{quantitative}\,\mathrm{trait} \end{array},$$

where *w* is an adjustable parameter, and we selected weight as 0.5 by default. *d*(*x*_*ij*_,*c*_*lj*_) represents the distance between *x*_*ij*_ and *c*_*lj*_. Let *x*_*ij*_ be the value of the *j*th phenotype in the *i*th accession, and *c*_*lj*_be the centroid of the *l*th cluster in the *j*th phenotype. The distance of the quantitative trait is measured by Euclidean distance *d*(*x*_*ij*_,*c*_*lj*_)

$$d\,\left(x_{i\,j},c_{l\,j}\right)={\left(x_{i\,j}-c_{l\,j}\right)}^{2}.$$

The qualitative trait is converted to dummy variable (0 or 1). For instance, a qualitative phenotype *Y* = {*Y*_1_,*Y*_2_,…,*Y*_*n*_} with *p* different trait levels is expanded to *p* indicator variables, *denotedasI*_A_(*y*)

$$I_{\text{A}}\,\left(y\right)=\{\begin{array}{c} \begin{array}{cc} 1, & \text{if}\,y_{\text{i}}\,\mathrm{equals}\,\mathrm{to}\,\mathrm{the}\,\mathrm{specific}\,\mathrm{level} \end{array}\\ \begin{array}{cc} 0, & \text{otherwise} \end{array} \end{array}.$$

Thus, Euclidean distance can be applied to measure the distance. The aim is to minimize the objective function *P*(*A*,*W*,*C*) during the procedure of iteration. To select the optimal number of clusters, we set criteria as follows: Shannon diversity index is above 90%, Nei’s diversity index is above 85%, and the proportion of variance explained is at least 70%.

### Modified Roger’s Distance

We proposed modified Roger’s distance to construct a CC, which is a small collection with minimal redundancy and maximal representative of the ES. The modified Roger’s distance is a suitable algorithm for mixed quantitative–qualitative phenotypes. The distance ($d_{E\,u\,l}^{2}$) for quantitative phenotype *j* (∈ *C*)can be expressed as

$$d_{E\,u\,l}^{2}=\sum_{j\in C}\sum_{i\neq i^{*}}{\left(x_{i\,j}-x_{i^{*}\,j}\right)}^{2},$$

where *C* is the quantitative phenotype, and *x*_*ij*_ and *x*_i^*j_ represent the value of the *i*th and *i*^∗^th accession in the *j*th trait, respectively. The distance ($d_{m\,R}^{2}$) for qualitative phenotype *j* (∈ *Q*) is defined as,

$$d_{m\,R}^{2}=\left\{ \begin{array}{c} 0,\mathrm{if}\,\mathrm{the}\,\mathrm{same}\,\mathrm{trait}\,\mathrm{level}\,\mathrm{between}\,i\,\mathrm{and}\,i^{*}\\ \sum_{j\in Q}\sum_{i\neq i^{*}}{\left(\log f_{i\,j}-\log f_{i^{*}\,\text{j}}\right)}^{2},\begin{array}{c} \mathrm{if}\,\mathrm{different}\,\mathrm{trait}\,\mathrm{level}\\ \mathrm{between}\,i\,\mathrm{and}\,i^{*} \end{array} \end{array}\right.\,$$

Where *Q* is the qualitative phenotype, and *f*_*ij*_ and *f*_i^*j_ represent the frequency of *i*th and *i*^∗^th accession in *j*th trait, respectively. Thus, the modified Roger’s distance can be of the form $d^{2}=d_{E\,u\,l}^{2}+d_{m\,R}^{2}$.

### Phenotypic Diversity

Phenotypic diversity is calculated using the Shannon–Weaver diversity index (*H*′) and Nei’s diversity index (Shannon and Weaver, 1962; Nei, 1973). The Shannon–Weaver diversity index (*H*′) is defined as

H=′∑i=1Spi⁢ln⁢(pi)ln⁢(S),

and Nei’s diversity index is defined as

$$1-\sum_{i=1}^{S}p_{i}^{2},$$

where *p*_*i*_ is the proportion of accessions in the *i*th cluster to the total number of germplasms and *S* is the total number of clusters. The value of *H*′ reflects the degree of evenness of germplasms. The higher *H*′, the more evenness. *H*′∈[0,1]. The value of Nei’s diversity index is bounded between 0 and ($1-\frac{1}{S}$), which really depends on the number of clusters.

### Assessment of the CC

To evaluate whether the CC is representative of the EC, five properties of the CC can be used to assess the characteristics and diversity structure of the traits. These properties include (1) the mean difference percentage ($\mathrm{MD}\%=\frac{1}{m}\,\sum_{i=1}^{m}\frac{\left|M_{\text{e}}-M_{\text{c}}\right|}{M_{\text{c}}}\times 100\%$), (2) the variance difference percentage ($\mathrm{VD}\%=\frac{1}{m}\,\sum_{i=1}^{m}\frac{\left|V_{\text{e}}-V_{\text{c}}\right|}{V_{\text{c}}}\times 100\%$), (3) the coincidence rate ($\mathrm{CR}\%=\frac{1}{m}\,\sum_{i=1}^{m}\frac{R_{\text{c}}}{R_{\text{e}}}\times 100\%$), (4) the variable rate ($\mathrm{VR}\%=\frac{1}{m}\,\sum_{i=1}^{m}\frac{C\,V_{\text{c}}}{C\,V_{\text{e}}}\times 100\%$), and (5) coverage ($\mathrm{Coverage}\%=\,\frac{1}{m}\,\sum_{i=1}^{m}\frac{D_{\text{c}}}{D_{\text{e}}}\times 100\%$), where *M, V, R, CV, D*, and *m* are the mean, variance, range, coefficient of variation, the number of clusters, and the number of traits, respectively. As for the subscript, *e* is short for the EC while *c* is short for the CC. We considered the CC to be representative of the EC if (1) MD% is small and the percentage of significant mean difference (α = 0.05) between the CC and the EC in all 29 phenotypic traits is no more than 20%, and (2) the CR% is greater than 80% (Hu et al., 2000; Kim et al., 2007).

Levene’s test was conducted, with 1,000 bootstrap iterations, to check homogeneity of variance among groups (Levene, 1960; Joseph et al., 2020). After testing for homogeneity of variance, we then checked the difference between the EC and the CC. For quantitative traits, traits with equal and unequal variances were, respectively, performed by Student’s *t*-test and Welch’s *t*-test (Delacre et al., 2017). For quantitative traits, Chi-squared test of homogeneity was performed to see whether germplasms of each phenotype was significant difference in distribution between the EC and the CC (Koehler and Wilson, 1986) (*p*-values < 0.05).

## Results

A total of 213 vegetable soybean germplasms were used to establish the CC for effective utilization. Thirteen redundant accessions were removed from the analysis, because these accessions, with different names or designators, have identical phenotypes in all traits to other varieties. There is no accession having missing values in all traits. After germplasm quality control, 200 accessions remained and regarded as entire collection (EC).

Our data suffered, to a certain extent, from missing rates, ranging from 0.5 to 26.5% (Supplementary Table 3). These missing phenotypes were estimated by chained equations in multiple imputation. The value of $\hat{\text{R}}\,$ statistic for each of the traits was smaller than 1.1 on the second-stage multiple imputation, indicating converged estimates on imputed phenotypes (Supplementary Table 4). Tables 1, 2 demonstrate behavioral patterns of central tendency and variability for quantitative traits and frequency distributions of trait characteristics for qualitative traits, respectively, for observed and imputed phenotypic traits. All imputed phenotypes, except stem color and plant type having slight differences, showed non-significant differences (*p*-values > 0.05) among observed and imputed phenotypic traits, suggesting reliable and accurate imputed values.

**TABLE 1:** Difference tests of quantitative traits between observed phenotypes and imputed phenotypes.

Phenotypic trait	Observed phenotypes		Imputed phenotypes^a^		Difference test (*p*-value)^b^
	*N*	Mean ± s.d.	*N*	Mean ± s.d.	
Seed length (mm)	200	8.96 ± 0.78	200	8.96 ± 0.78	1.00
Seed width (mm)	200	8.28 ± 0.59	200	8.28 ± 0.59	1.00
Seed thickness (mm)	200	7.09 ± 0.67	200	7.09 ± 0.67	1.00
100 seed weight (g)	200	33.41 ± 7.51	200	33.41 ± 7.51	1.00
Plant height (cm)	198	37.09 ± 10.71	200	37.01 ± 10.69	0.94
Leaflet length (cm)	200	10.6 ± 7.61	200	10.6 ± 7.61	1.00
Leaflet width (cm)	200	7.22 ± 1.18	200	7.22 ± 1.18	1.00
Pod length (cm)	149	4.59 ± 0.53	200	4.56 ± 0.5	0.62
Pod width (cm)	149	1.22 ± 0.28	200	1.21 ± 0.24	0.71
Stem length to first pod (cm)	150	11.85 ± 4.41	200	11.4 ± 4.3	0.34
Shelling rate (%)	150	56.79 ± 6.97	200	57.02 ± 6.48	0.75
Immature seed length (mm)	149	15.49 ± 1.63	200	15.61 ± 1.61	0.51
Immature seed width (mm)	150	11.04 ± 3.13	200	11.03 ± 3.49	0.96
Immature seed thickness (mm)	150	8.07 ± 0.82	200	8.11 ± 0.85	0.67
100 immature seed weight (g)	149	69.05 ± 14.87	200	69.57 ± 13.74	0.74

*N, number of germplasms; s.d., standard deviation. ^*a*^Multiple imputation was used to estimate missing phenotypes. ^*b*^Student’s t-test was conducted to test difference among observed and imputed phenotypes.*

**TABLE 2:** Difference tests of qualitative traits between observed phenotypes and imputed phenotypes.

Phenotypic trait	Observed phenotypes	Imputed phenotypes^a^	Difference test (*p*-value)^b^
	*N* (%)	*N* (%)	
Seed shape			1.00
Round	107 (53.5)	107 (53.5)	
Oblate	21 (10.5)	21 (10.5)	
Oval	58 (29)	58 (29)	
Flat	13 (6.5)	14 (7)	
Seed coat color			1.00
Yellowish white	26 (13)	28 (14)	
Yellow	69 (34.5)	73 (36.5)	
Green	92 (46)	97 (48.5)	
Pale brown	1 (0.5)	1 (0.5)	
Reddish brown	1 (0.5)	1 (0.5)	
Hilum color			0.37
Light yellow	13 (6.5)	22 (11)	
Yellow	58 (29)	60 (30)	
Brown	102 (51)	109 (54.5)	
Green	4 (2)	9 (4.5)	
Hypocotyl coloration			1.00
Green	131 (65.5)	132 (66)	
Purple	68 (34)	68 (34)	
Stem color			0.01
Light green	106 (53)	106 (53)	
Green	68 (34)	68 (34)	
Dark green	8 (4)	26 (13)	
Number of branches			1.00
Low	58 (29)	58 (29)	
Medium	79 (39.5)	80 (40)	
High	62 (31)	62 (31)	
Leaflet size			0.66
Small	81 (40.5)	102 (51)	
Medium	58 (29)	78 (39)	
Large	11 (5.5)	20 (10)	
Leaflet shape			0.98
Lanceolate	1 (0.5)	1 (0.5)	
Lanceolate to oblong	37 (18.5)	53 (26.5)	
Rhomboid	24 (12)	36 (18)	
Oval	46 (23)	62 (31)	
Elliptic	39 (19.5)	48 (24)	
Leaf color			0.52
Green	46 (23)	69 (34.5)	
Dark green	104 (52)	131 (65.5)	
Plant type			**0.04**
Determinate	142 (71)	177 (88.5)	
Semi-determinate	7 (3.5)	23 (11.5)	
Pubescence density			1.00
Absent	4 (2)	5 (2.5)	
Rare	20 (10)	20 (10)	
Sparse	33 (16.5)	33 (16.5)	
Medium	86 (43)	86 (43)	
Dense	56 (28)	56 (28)	
Pubescence color			1.00
Grayish white	102 (51)	104 (52)	
Pale brown	63 (31.5)	64 (32)	
Brown	32 (16)	32 (16)	
Corolla color			1.00
White	138 (69)	139 (69.5)	
Purple throat	34 (17)	34 (17)	
Purple	27 (13.5)	27 (13.5)	
Pod set capacity			0.95
Low	13 (6.5)	17 (8.5)	
Medium	54 (27)	76 (38)	
High	82 (41)	107 (53.5)	

*N, number of germplasms;%, percentage. ^*a*^Multiple imputation was used to estimate missing phenotypes. ^*b*^Chi-squared test was conducted to test difference among observed and imputed phenotypes. Bold value (p-value < 0.05) represents significant difference between two sets.*

**FIGURE 1:**
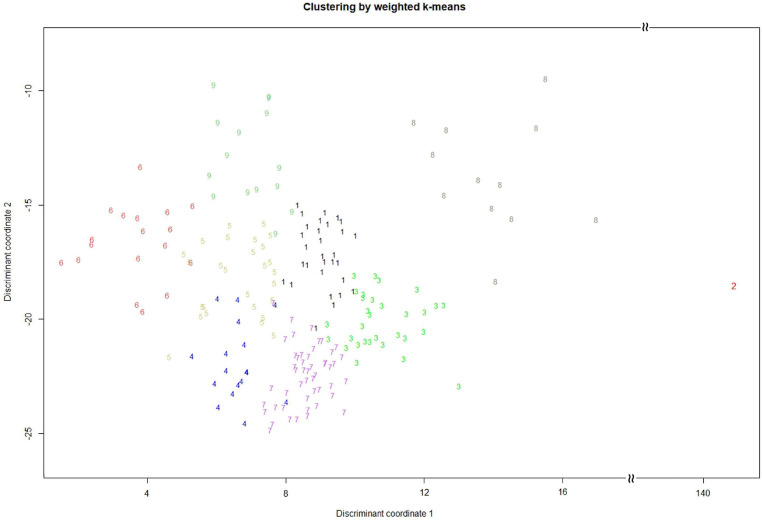
Cluster analysis by weighted *k*-means for mixed quantitative–qualitative phenotypic traits. The vegetable soybean germplasms (200 accessions) are separately grouped into nine clusters.

We conducted weighted *k*-means clustering method, using completed phenotypes (i.e., observed plus imputed values on 29 mixed quantitative–qualitative phenotypic traits), to search for the population structure of the whole picture on the EC. Figure 1 demonstrates that our vegetable germplasms can be grouped into nine clusters, using weighted *k*-means clustering algorithm for 29 mixed-type phenotypic traits. We calculated modified Roger’s distance for mixed quantitative–qualitative phenotypic traits to capture pairwise genetic distance among accessions in the EC. The larger genetic distance, the much dissimilar. As a result, 30 accessions (Table 3 and Supplementary Material 1) were selected from the EC and regarded as CC, showing maximal genetic distance among accessions. Levene’s test was first applied to examine homogeneity of variances for each of phenotypic traits, followed by the difference test to compare whether there is difference between the CC and the EC. Our results showed that no significant difference (*p*-values > 0.05) was observed between the CC and the EC in all traits, except plant height (Tables 4, 5), indicating that the central tendency and variability of phenotypic traits in the CC were consistent with the EC.

**TABLE 3:** Selected core collection of vegetable soybean.

Germplasm ID	Accession name^a^	Origin	Germplasm ID	Accession name^a^	Origin
KG0164	Nuli-6-G2657	Unknown	KG0127	207	China
KG0038	G10502	Japan	KG0180	Hsueh Chih Hsia-28	Japan
KG0031	G10493	Japan	KG0169	KS1822	Taiwan
KG0009	ESB-67-9 [KG0009]	Taiwan	KG0175_1	Kahori	Japan
KG0153	KVS39	Taiwan	KG0163	D-62-7815	United States
KG0137	G7332	United States	KG0181	Hsueh Chih Hsia-34	Japan
KG0011	ESB-67-14	Taiwan	KG0179	Hsueh Tou	Unknown
KG0159	Kaorihime	Japan	KG0177	GC 84136-P-4-1-8	Taiwan
KG0101	Ryokukou [KG0101]	Japan	KG0199	Goku Wase Osuro [KG0199]	Japan
KG0125	Chen Hsiang	Taiwan	KG0172	Lu Kuang-75	Japan
KG0185	AGS186	Taiwan	KG0171	Lu Kuang-74	Japan
KG0192	Sakata Kairyo Mikowashima	Japan	KG0113	Taiuasu Dare	Japan
KG0106	Kamui	Japan	KG0132	Mainland China	Hong Kong
KG0196	Tzuraroko Daizu	Japan	KG0010	ESB-67-10	Taiwan
KG0050	Fubaye	Unknown	KG0162	G10137	Philippines

*^*a*^The 30 accessions were selected, using modified Roger’s distance, as the core collection.*

**TABLE 4:** Difference test of quantitative traits between the core collection and the entire collection in vegetable soybean.

Phenotypic trait	Entire collection (EC)							Core collection (CC)^a^							Difference test^b^	
	*N*	Min	Max	Range	Mean	*SD*	CV (%)	*N*	Min	Max	Range	Mean	*SD*	CV (%)	p.homo	p.diff
Seed length (mm)	200	7.1	11.2	4.1	9.0	0.8	8.7	30	7.2	10.8	3.6	9.2	1.2	12.8	<0.001	0.40
Seed width (mm)	200	6.2	9.7	3.5	8.3	0.6	7.1	30	6.3	9.7	3.4	8.3	0.8	9.3	0.047	0.88
Seed thickness (mm)	200	5.1	8.8	3.7	7.1	0.7	9.5	30	5.1	8.8	3.7	6.9	0.8	11.4	0.25	0.22
100 seed weight (g)	200	4.2	51.2	47.0	33.4	7.5	22.5	30	4.2	51.2	47.0	32.4	12.6	38.9	<0.001	0.68
Plant height (cm)	198	17.3	70.7	53.4	37.1	10.7	28.9	30	17.3	70.7	53.4	45.2	14.4	31.9	0.007	**0.01**
Leaflet length (cm)	200	7.0	116.0	109.0	10.6	7.6	71.8	30	7.4	116.0	108.6	14.0	19.3	138.3	0.11	0.08
Leaflet width (cm)	200	1.4	10.4	9.0	7.2	1.2	16.3	30	1.4	8.8	7.4	7.3	1.5	20.9	0.52	0.69
Pod length (cm)	149	1.3	5.8	4.5	4.6	0.5	11.6	30	1.3	5.8	4.5	4.5	0.8	18.7	0.01	0.55
Pod width (cm)	149	0.9	4.3	3.4	1.2	0.3	23.0	30	0.9	4.3	3.4	1.3	0.6	44.3	0.10	0.19
Stem length to first pod (cm)	150	4.2	22.3	18.1	11.9	4.4	37.2	30	4.3	22.3	18.0	13.6	5.0	36.9	0.24	0.052
Shelling rate (%)	150	4.8	75.0	70.2	56.8	7.0	12.3	30	4.8	75.0	70.2	54.2	11.5	21.1	0.10	0.10
Immature seed length (mm)	149	6.0	18.1	12.1	15.5	1.6	10.5	29	6.0	18.0	12.0	15.4	2.4	15.7	0.20	0.75
Immature seed width (mm)	150	1.1	40.9	39.8	11.0	3.1	28.4	30	1.1	40.9	39.8	12.2	6.2	51.2	0.04	0.34
Immature seed thickness (mm)	150	5.8	9.7	3.9	8.1	0.8	10.2	30	5.8	9.7	3.9	8.0	0.9	11.3	0.91	0.83
100 immature seed weight (g)	149	6.2	100.0	93.8	69.1	14.9	21.5	30	6.2	100.0	93.8	64.7	24.7	38.1	< 0.001	0.36

*N, number of germplasms; SD, standard deviation; CV, coefficient of variation; p.homo, p-value of homogeneity test for variance (Levene’s test); p.diff, p-value of difference test (Student’s t-test or Welch’s t-test). ^*a*^Core collection was identified using modified Roger’s distance for mixed quantitative–qualitative phenotypic traits. ^*b*^Student’s t-test (if assumption of homogeneity of variance is met) and Welch’s t-test (if assumption of homogeneity of variance is not met) were used to conduct mean difference among two collections. Bold value (p-value < 0.05) represents significant difference between two sets.*

**TABLE 5:** Difference test of qualitative traits between the core collection and the entire collection in vegetable soybean.

Phenotypic trait	Entire collection (EC)	Core collection (CC)^a^	Difference test (*p*-value)^b^
	*N* (%)	*N* (%)	
Seed shape			0.91
Round	107 (53.5)	15 (50.0)	
Oblate	21 (10.5)	3 (10.0)	
Oval	58 (29.0)	9 (30.0)	
Flat	13 (6.5)	3 (10.0)	
Seed coat color			0.97
Yellowish white	26 (13.0)	4 (14.3)	
Yellow	69 (34.5)	9 (32.1)	
Green	92 (46.0)	15 (53.6)	
Pale brown	1 (0.5)	0 (0)	
Reddish brown	1 (0.5)	0 (0)	
Hilum color			0.28
Light yellow	13 (6.5)	2 (7.7)	
Yellow	58 (29.0)	11 (42.3)	
Brown	102 (51.0)	11 (42.3)	
Green	4 (2.0)	2 (7.7)	
Hypocotyl coloration			0.68
Green	131 (65.5)	18 (60.0)	
Purple	68 (34.0)	12 (40.0)	
Stem color			0.49
Light green	106 (53.0)	19 (63.3)	
Green	68 (34.0)	11 (36.7)	
Dark green	8 (4.0)	0 (0)	
Number of branches			0.77
Low	58 (29.0)	9 (30.0)	
Medium	79 (39.5)	10 (33.3)	
High	62 (31.0)	11 (36.7)	
Leaflet size			0.64
Small	81 (40.5)	10 (43.5)	
Medium	58 (29.0)	11 (47.8)	
Large	11 (5.5)	2 (8.7)	
Leaflet shape			0.17
Lanceolate	1 (0.5)	1 (4.2)	
Lanceolate to oblong	37 (18.5)	7 (29.2)	
Rhomboid	24 (12.0)	1 (4.2)	
Oval	46 (23.0)	11 (45.8)	
Elliptic	39 (19.5)	4 (16.6)	
Leaf color			0.75
Green	46 (23.0)	6 (25.0)	
Dark green	104 (52.0)	18 (75.0)	
Plant type			0.80
Determinate	142 (71.0)	22 (91.7)	
Semi-determinate	7 (3.5)	2 (8.3)	
Pubescence density			0.10
Absent	4 (2.0)	2 (6.7)	
Rare	20 (10.0)	6 (20.0)	
Sparse	33 (16.5)	4 (13.3)	
Medium	86 (43.0)	7 (23.3)	
Dense	56 (28.0)	11 (36.7)	
Pubescence color			0.46
Grayish white	102 (51.0)	11 (39.3)	
Pale brown	63 (31.5)	11 (39.3)	
Brown	32 (16.0)	6 (21.4)	
Corolla color			0.65
White	138 (69.0)	20 (66.7)	
Purple throat	34 (17.0)	7 (23.3)	
Purple	27 (13.5)	3 (10.0)	
Pod set capacity			0.68
Low	13 (6.5)	1 (4.2)	
Medium	54 (27.0)	8 (33.3)	
High	82 (41.0)	15 (62.5)	

*N, number of germplasms; %, percentage. ^*a*^Core collection was identified using modified Roger’s distance for mixed quantitative–qualitative phenotypic traits. ^*b*^Chi-squared test was conducted to test for difference among two collections.*

Table 6 showed cluster and diversity comparison between the CC and the EC for individual traits in vegetable soybean. The Shannon–Weaver diversity index of individual phenotypic traits in the CC and the EC ranged from 0.68 to 0.98 and from 0.64 to 0.97 in quantitative traits and ranged from 0.41 to 1.00 and from 0.27 to 0.99 in qualitative traits, with an overall average of 0.88 and 0.83, respectively. Similarly, Nei’s diversity index in the CC and the EC ranged from 0.46–0.89 and 0.50–0.89 in quantitative traits and ranged from 0.15 to 0.75 and from 0.09 to 0.74, with an overall average of 0.66 and 0.66, respectively. Compared to the EC, our CC suggested that approximately 79.31% (23 out of 29) of phenotypic traits demonstrated higher or equal diversities on Shannon–Weaver diversity and/or Nei’s diversity indices, indicating that about 80% phenotypic diversities were retained. It is worth noticing that three traits (100 seed weight, pod width, and immature seed width) in the CC demonstrated more evenness than those in the EC, resulting in increase in diversities in spite of losing clusters. About 21.69% (6 out of 29) of phenotypic traits demonstrated a mild degree (2–9%) of diversity lost in the CC due to the loss of clusters and/or reduced evenness among clusters. Taking all 29 phenotypic traits together, a high overall diversity (Shannon–Weaver diversity index is 0.91, Nei’s diversity index is 0.85) was observed in the EC, and a reasonably high overall diversity (Shannon–Weaver diversity index is 0.83, Nei’s diversity index is 0.81) was retained in the CC (data not shown), showing a mild degree (4–8%) of overall diversity lost in the CC. This suggested that our selected CC is representative of diversity from the EC.

**TABLE 6:** Diversity comparison between the core collection and the entire collection in vegetable soybean.

Phenotypic trait	Clusters and diversity					
	Entire collection (EC)			Core collection (CC)^a^		
	*k*_EC_^b^	*H*′	Nei’s	*k*_CC_	*H*′	Nei’s
**Quantitative traits**						
Seed length (mm)	6	0.91	0.78	6	0.97	0.82
Seed width (mm)	9	0.94	0.86	9	0.95	0.86
Seed thickness (mm)	5	0.95	0.77	5	0.98	0.79
100 seed weight (g)	12	0.93	0.89	11	0.95	0.89
Plant height (cm)	11	0.95	0.89	9	0.89	0.83
Leaflet length (cm)	3	0.65	0.50	3	0.68	0.46
Leaflet width (cm)	6	0.91	0.79	6	0.85	0.74
Pod length (cm)	10	0.86	0.84	7	0.89	0.79
Pod width (cm)	5	0.64	0.54	4	0.86	0.66
Stem length to first pod (cm)	5	0.96	0.77	4	0.99	0.74
Shelling rate (%)	5	0.88	0.75	4	0.90	0.69
Immature seed length (mm)	6	0.89	0.78	6	0.91	0.78
Immature seed width (mm)	9	0.86	0.83	8	0.97	0.86
Immature seed thickness (mm)	6	0.97	0.82	5	0.95	0.77
100 immature seed weight (g)	6	0.84	0.75	6	0.95	0.81
**Qualitative traits**						
Seed shape	4	0.80	0.61	4	0.84	0.64
Seed coat color	5	0.65	0.61	3	0.89	0.59
Hilum color	4	0.69	0.55	4	0.81	0.63
Hypocotyl coloration	2	0.93	0.45	2	0.97	0.48
Stem color	3	0.75	0.52	2	0.95	0.46
Number of branches	3	0.99	0.66	3	1.00	0.66
Leaflet size	3	0.81	0.55	3	0.84	0.57
Leaflet shape	5	0.87	0.74	5	0.80	0.67
Leaf color	2	0.89	0.43	2	0.81	0.38
Plant type	2	0.27	0.09	2	0.41	0.15
Pubescence density	5	0.82	0.70	5	0.92	0.75
Pubescence color	3	0.91	0.60	3	0.97	0.65
Corolla color	3	0.75	0.47	3	0.76	0.49
Pod set capacity	3	0.83	0.56	3	0.72	0.50

*k_*EC*_, number of clusters in the entire collection; k_*CC*_, number of clusters in the core collection; H′, Shannon–Weaver diversity index; Nei’s, Nei’s diversity index. ^*a*^Core collection was identified using modified Roger’s distance for mixed quantitative–qualitative phenotypic traits. ^*b*^Clustering analyses were conducted using weighted k-means clustering algorithm.*

The CC selected by modified Roger’s distance gave a small value of MD% (6.35%), an intermediate value of VD% (52.90%), a large value of CR% (97.70%), a very high value of VR% (149.80%), and a very high percentage of coverage (94.76% for qualitative traits, 90.38% for quantitative traits, and 92.5% for the combined) (Table 7). These properties of the CC suggested that the CC retained valuable characteristics and large variability for phenotypic traits in the EC. In addition, the percentage of significant difference was 3.45%, which is less than the threshold of 20%, indicating a very low significant difference between the EC and the CC in traits. This suggested that the CC selected by the modified Roger’s distance was representative of the EC.

**TABLE 7:** Evaluation in percentage of the trait differences between the core collection and the entire collection in vegetable soybean.

Modified Roger’s distance	MD%	VD%	CR%	VR%	Coverage %^a^		
					Quantitative traits	Qualitative traits	Combined traits
The property of the CC	6.35	52.90	97.70	149.80	90.38	94.76	92.50
Percentage of significant difference^b^	3.45						

*MD%, mean difference percentage; VD%, variance difference percentage; CR%, coincidence rate; VR%, variable rate. ^*a*^Coverages were computed according to quantitative traits, qualitative traits, and combined traits. ^*b*^The core collection is considered to be representative of the entire collection if (1) MD% is small and percentage of significant mean difference (α = 0.05) between the core collection and the entire collection in all 29 phenotypic traits is no more than 20%, and (2) the CR% is greater than 80%.*

## Discussion

To the best of our knowledge, our vegetable soybean germplasms consisted of 213 accessions, which are the largest collection worldwide. Vegetable soybean becomes popular just for more than one decade and mostly in East Asia, e.g., Japan, Taiwan, China, and South Korea; therefore, other countries take merely a small amount of production; the majority of vegetable soybeans are imported from Asia (Wang, 2018; Carneiro et al., 2020). This is why it did not attract the attention of breeders until this century. Hence, there are only limited accessions available in the seedbank. The same results are also shown in this study. Therefore, without special attention and care, the vegetable germplasm may not be focused. The market determines the importance of vegetable soybean and breeder’s attention.

In this study, a total of 29 traits (15 quantitative and 14 qualitative traits) were selected from 47 phenotypic traits, based on at most 30% threshold of missing rate. Among them, 5 traits had no missing rates, 10 traits had low missing rates (0.5–11.5%), and the remaining traits had moderate missing rates (25.5–26.5%) (Supplementary Table 3). Generally, missing phenotypes are very often seen in many germplasms. Fortunately, our phenotypes did not seriously suffer from missing data. However, missing values in phenotypes, even at a low or moderate level, still leave space for uncertainty in search for the behavior of traits in vegetable soybean germplasms.

Phenotype completeness is the key to access in developing a core set of germplasm for efficient exploration and effective utilization in breeding programs. In fact, the impact of missing data on phenotypic traits research can be serious, and the utilization of phenotypes is often limited by incomplete phenotypes of interest (Haupt and Schmid, 2020). In particular, trait-based analysis like CC really relies on the completeness of phenotypes. Hence, a model-based imputation algorithm, such as multiple imputation by chained equations, can fill the gap in the trait-based germplasm database. As discussed by Taugourdeau et al. (2014), multiple imputation generally has accurate estimates on missing phenotypes and stable convergence statistic $\hat{\text{R}}$s, which were smaller than 1.1 (Supplementary Table 4), suggesting that multiple imputation can produce stable and reliable estimates for missing phenotypes. It is worth noticing that accessions for diverse landraces or genotypes from different countries or continents may demonstrate diverse phenotypes in traits, indicating that some of these traits may have unbalanced distributions. This means that the distributions of the phenotypes for some traits are unbalanced (i.e., skewed distributions). For instance, for some traits such as 100 seed weight (g), 100 immature seed weight (g), and shelling rate (%), most values were high, but with few extreme low values; similarly, for plant height (cm), leaflet length (cm), and immature seed width (mm), most values were low, but with few extreme high values. Hence, multiple imputation in chained equations is an appropriate method to produce accurate and robust results for the unbalanced traits (Taugourdeau et al., 2014). Multiple imputation has addressed some imputation problems, including structural (multi)collinearity, estimation convergence, semi-continuous data analysis, and data separation, which were neglected by other approaches (Su et al., 2011). Therefore, the multiple imputation method is one of the best methods for traits imputation of missing data, with a maximum tolerance of 30% missing rate. Fortunately, our vegetable germplasm has a low level of missing data, and our imputed missing data exhibited converged estimates as well as a lower error on the estimation of missing phenotypic trait values.

The selection of sampling strategies does affect the results of the selection of the CC, as well as its diversity. To have a better illustration, we compared the proposed modified Roger’s distance algorithm with other sampling schemes, including stratified proportional sampling (SPS), simple random sampling (SRS), and advanced M strategy (using PowerCore software). As the difference test, there were only two traits, seed width by advanced M strategy and number of branches by SRS, showing significant mean difference between the EC and the CC (Table 5 and Supplementary Table 5). Most of the tests were found to be non-significant between the EC and the CC, indicating that four sampling methods had similar abilities to collect the CC. Supplementary Table 6 demonstrates the phenotypic diversity using three other sampling strategies. Among the four strategies, the CC from modified Roger’s distance algorithm showed the highest phenotypic diversity.

The CC collected from distinct sampling strategies was evaluated. We evaluated the percentage of the trait differences between the CC and the EC, using PowerCore, SPS, and SRS methods, compared to our developed modified Roger’s distance (please refer to Table 7 and Supplementary Table 7). All methods showed a very small mean difference and a very low percentage of significant difference (MD% < 20%) between the CC and the EC, demonstrating equal central tendency properties. Comparing coincidence rates, the modified Roger’s distance had the highest CR% = 97.7% (>80%), followed by Advanced M strategy by PowerCore (CR% = 96.56%), but SRS (CR% = 69.18) and SPS (CR% = 58.38%) had poor genetic variability for traits. Our modified Roger’s distance (VD% = 52.90%, VR% = 149.80%) and PowerCore (VD% = 52.16%, VR% = 151.68%) showed equally slightly larger variance difference percentage and larger variable rate, suggesting to provide a good representation of the genetic diversity of the EC. However, SRS had a small VD% (41.94%) and an intermediate value of VR% (117.41%); SPS demonstrated an extremely large VD% (356.85%) and extremely low VR% (89.67%). This indicated that the properties of the CC constructed by both SPS and SRS were not representative of the EC. Furthermore, we found that the modified Roger’s distance demonstrated the highest coverage% in quantitative (90.38%), qualitative (94.76%), and combined traits (92.50%), compared to SPS and SRS. Notice that PowerCore had 100% coverage percentage in qualitative traits, but not available in quantitative traits and the combined traits. As discussed above, different sampling strategies can affect the properties of the CC. Therefore, the CC constructed by the modified Roger’s distance can capture accessions with valuable and/or special characteristics and larger variability from the EC.

In this study, a total of 30 accessions were selected as our CC. Investigating origins and major features of the CC indicate that these accessions are excellent germplasms with abundant genetic diversity, including several germplasm test lines from the vegetable soybean breeding programs in Taiwan, plenty of germplasms introduced from Japan, some Japanese varieties that have long been planted in Taiwan, and other germplasms that come from United States, China, Hong Kong, Philippines, and other unknown countries (Tables 3–7). As for the important traits of vegetable soybean (e.g., seed size and weight, cold tolerance, isoflavone content, and yield), most accessions in CC cover all the range of these traits to be the same as or approximately similar to that of the EC, suggesting that our CC can be representative of the diversity of the EC. For example, immature seed size and weight were found to be associated with yield, quality, and appearance for vegetable soybean (Krisnawati and Adie, 2015). The two most important vegetable soybean cultivars in Taiwan are Kaohsiung 9 and Kaohsiung 12, which are characterized by 100 immature seed weight (81 g for #9 and 75 g for #12) and shattering rate (61% for #9 and 48% for #12). Our CC revealed that 100 immature seed weight and shattering rate ranged between 37 and 100 g and between 41 and 75%, respectively. In addition, our CC includes two small seed varieties (KG0050: 6.8 g, KG0127: 6.2 g). These suggested that our CC provides potential for breeding programs. On the other hand, pod set capacity affects the production of vegetable soybean at low temperature (Kurosaki et al., 2003). Among the CC, all three levels of pod set capacity were covered and most accessions have high pod set capacity (Table 5), which reveals that our accessions in CC may have potential for cold tolerance. It indicated that the CC contained market-oriented phenotypes, and it can be used as a priority resource for vegetable soybean breeding applications. In the future, if there are any requirements for the introductions of the novel disease/pest resistances or stress tolerances, this CC can be applied first for the breeding programs. Taken together, the CC determined by the new algorithm not only retained most diversity from the EC but also will be useful for the breeders and plant scientists for breeding program.

From diversity investigation and assessment of the CC (please refer to Tables 4–7), the CC retained valuable characteristics and large variability for phenotypic traits in the EC, suggesting representativeness of the whole germplasms. We take the edamame traits of the immature seeds as an example. There are four traits (immature seed length, immature seed width, immature seed thickness, and 100 immature seed weight) related to immature seeds. From Table 4, we can see that the ranges (includes minimal and maximum) of the four traits in the CC fully covered those in the EC, with quite similar means. From the difference test, we can also obtain non-significant difference among the CC and the EC, suggesting that the CC has high coverage to the EC.

Among the CC, there is one germplasm called Mikawashima. It is a local variety in Japan, which was bred through a variety of improvements using a landrace by the Sakata Seed Company in Japan (Mimura et al., 2007). It might contain several common and popular traits of vegetable soybean in Japan but had not been applied by Taiwanese breeders. Therefore, Mikawashima became an outlier in the clustering analysis (Figure 1).

There are some limitations of this study. First, many of the quantitative phenotypic traits are influenced by environmental effects. To minimize environmental effects, we only focused on vegetable soybean accessions planted in autumn to remove possible effects from environmental factors. In addition, our collected meteorology data showed that environmental factors did not significantly (*p*-values > 0.05) affect our trait values measured during 1995–1998 autumn seasons (Supplementary Table 2). Second, the CC was established by using trait-based data. However, some studies suggested that the choice of data type (phenotypes or genotypes) is not the main cause to alter the diversity (Grenier et al., 2000; Yun et al., 2015). The quality of data control is the first thing taken into consideration regardless of the data used for developing the CC. In the current study, we have conducted data quality control, including focusing on autumn crops and redundant exclusion, to remove possible noises and biases and produce reliable results.

The CC accounted for 15% (30/200) of the entire collection. Moreover, the high diversity of the CC indicated that the germplasms were simplified without losing information. The result showed that the modified Roger’s distance algorithm was a good approach to construct the CC. This study provides a detailed list of the CC, which can be a basis for future research of vegetable soybeans and breeding practices.

## Conclusion

To the best of our knowledge, this study reported results of the first CC for vegetable soybean (or edamame) worldwide. This study consisted of the largest vegetable soybean germplasms worldwide at present, which provided valuable and/or special information on Taiwanese vegetable soybean germplasms. We proposed a modified Roger’s distance algorithm, which is a mixed-type sampling strategy, to construct a CC composed of a total of 30 accessions based on morphological traits. It was evident that the properties of the CC constructed by the modified Roger’s distance algorithm were reliable, stable, and feasible. With the representativeness of the whole germplasms, the high-quality CC possessed small mean difference, high coincidence rate, variable rate, and high coverage. The CC preserved the high phenotypic diversity in germplasms of vegetable soybean. The removal of redundancy enables us to make full use of the vegetable soybean germplasms for overall phenotype clustering. With the support of the material, the CC can be helpful for further breeding of commercial varieties.

## Data Availability Statement

The original contributions generated for this study are included in the article/Supplementary Material, further inquiries can be directed to the corresponding author.

## Author Contributions

C-FK: study conception and design and acquisition and analysis of data. C-FK, S-SH, C-SW, D-GL, and SC: interpretation of data. C-FK and S-SH: drafting the manuscript. C-FK and Z-YL: manuscript revision. All authors read and approved the final manuscript.

## Conflict of Interest

The authors declare that the research was conducted in the absence of any commercial or financial relationships that could be construed as a potential conflict of interest.

## Supplementary Material

Supplementary Table 1 Forty-seven phenotypic traits of vegetable soybean in Taiwan.

Supplementary Table 2 Meteorology data during 2006–2009 in Kaohsiung, Taiwan.

Supplementary Table 3 Summary information of missing rate in phenotypic traits.

Supplementary Table 4 Summary results of imputed phenotypic traits by chained equation in multiple imputation.

Supplementary Table 5 Difference test of phenotypic traits between the core collection (using PowerCore, SPS, and SRS methods) and the entire collection in vegetable soybean.

Supplementary Table 6 Diversity between the core collection (using PowerCore, SPS and SRS methods) and the entire collection in vegetable soybean.

Supplementary Table 7 Evaluation of the core collection (using PowerCore, SPS, and SRS methods) in percentage of the trait differences with the entire collection in vegetable soybean.

Supplementary Material 1 The raw data of the core collection in vegetable soybean.
