# Supplementary material for: A Modified Roger’s Distance Algorithm for Mixed Quantitative–Qualitative Phenotypes to Establish a Core Collection for Taiwanese Vegetable Soybeans
Source: Front Plant Sci. 2021 Jan 12;11:612106. doi: 10.3389/fpls.2020.612106 (PMC7835400; doi:10.3389/fpls.2020.612106)
Supplement: Supplementary Table 1 — Forty-seven phenotypic traits of vegetable soybean in Taiwan. [file Table_1.DOCX]

**Supplementary Table 1.** Forty-seven phenotypic traits of vegetable soybean in Taiwan

| **Category** | **Phenotypic traits^a^** | |
| --- | --- | --- |
| Morphology | Seed length (mm)  Seed width (mm)  Seed thickness (mm)  Leaflet length (cm)  Leaflet width (cm)  Pod length (cm)  Pod width (cm)  Single pod weight (g)  Number of pods per 500 g  Number of seeds per pod  Shelling rate (%)  Immature seed length (mm)  Immature seed width (mm)  Immature seed thickness (mm)  Seed shape  Seed color  Hilum color  Hypocotyl coloration  Number of nodes on main stem | Stem color  Number of branches  Leaflet size  Leaflet shape  Number of leaflets  Leaf color  Pubescence  Pubescence color  Corolla color  Pod set capacity  Pod length  Pod width  Pod shape  Pod color  Immature seed size  Immature seed coat color  Immature seed texture  Easiness of pod removal  Storability |
| Growth | Plant height (cm)  Internode length (cm)  Stem length to first pod (cm)  Plant type  Lodging score |  |
| Phenology | From sowing to flowering (days)  From bloom to harvest (days) |  |
| Production | 100 seed weight (g)  100 immature seed weight (g) |  |
| ^a^Phenotypic traits were investigated at Kaohsiung District Agricultural Research and Extension Station, COA, followed the guidelines of distinctness, uniformity and stability (DUS) test for vegetable soybean. Each accession was characterized for 47 phenotypic traits in relation to morphology (38 traits), growth (5 traits), phenology (2 traits) and production (2 traits). | | |

**Supplementary Table 2.** Meteorology data during 1995-1998 in Kaohsiung, Taiwan

| **Meteorology^a^** | **Mean ± s.d.^c^** | | | |  | **ANOVA**  **p-value^b^** | |
| --- | --- | --- | --- | --- | --- | --- | --- |
|  | **1995** | **1996** | **1997** | **1998** |  | **Oct.-Nov.**  **(Autumn)** | **Jan.-Dec.**  **(Whole year)** |
| **Kaohsiung District Agricultural Research and Extension Station** | | | | |  |  |  |
| Average temperature (℃) | 24.23±3.83 | 24.42±3.70 | 24.59±3.36 | 25.55±3.00 |  | 0.93 | 0.79 |
| Average relative humidity (%) | 74.48±3.63 | 76.40±3.59 | 75.29±3.59 | 74.95±2.59 |  | 0.20 | 0.57 |
| Duration of sunshine (hours) | 153.48±25.18 | 158.13±42.23 | 136.08±39.19 | 160.67±33.74 |  | 0.35 | 0.34 |
| Precipitation (Mm) | 95.71±126.05 | 166.96±212.50 | 262.08±349.75 | 237.29±311.06 |  | 0.57 | 0.42 |
| **Kaohsiung Weather Station** | | | | |  |  |  |
| Average temperature (℃) | 25.66±3.28 | 25.45±3.32 | 25.13±3.79 | 25.36±3.65 |  | 0.92 | 0.99 |
| Average relative humidity (%) | 75.91±4.27 | 75.17±3.66 | 75.17±5.09 | 74.50±3.87 |  | 0.59 | 0.88 |
| Duration of sunshine (hours) | 188.43±19.34 | 197.08±41.09 | 190.21±31.60 | 210.26±27.77 |  | 0.67 | 0.31 |
| Precipitation (Mm) | 185.95±287.78 | 96.47±107.15 | 139.16±201.10 | 159.66±275.14 |  | 0.35 | 0.84 |
| Days with precipitation (days) | 7.36±4.61 | 8.82±6.38 | 8.73±6.15 | 6.55±5.75 |  | 0.59 | 0.75 |
| Abbreviation: s.d., standard deviation.  ^a^The meteorology data were recorded and collected per day during 1995-1998 separately by the Kaohsiung District Agricultural Research and Extension Station and the Kaohsiung Weather Station of the Central Weather Bureau in Taiwan. ^b^One-way ANOVA was conducted to test for differences among four years, followed by paired samples *t*-test only when ANOVA p-value reached significant difference. ^c^Means followed by the same letter(s) were not significantly difference (p-value<0.05) as determined by Bonferroni correction method. | | | | | | | |

**Supplementary Table 3.** Summary information of missing rate in phenotypic traits

| **Phenotypic trait** | **N** | **Missing rate (%)^a^** |
| --- | --- | --- |
| **Quantitative traits:** |  |  |
| Seed length (mm) | 200 |  |
| Seed width (mm) | 200 |  |
| Seed thickness (mm) | 200 |  |
| 100 seed weight (g) | 200 |  |
| Plant height (cm) | 198 | 1.0 |
| Leaflet length (cm) | 200 |  |
| Leaflet width (cm) | 200 |  |
| Pod length (cm) | 149 | 25.5 |
| Pod width (cm) | 149 | 25.5 |
| Stem length to first pod (cm) | 150 | 25.0 |
| Shelling rate (%) | 150 | 25.0 |
| Immature seed length (mm) | 149 | 25.5 |
| Immature seed width (mm) | 150 | 25.0 |
| Immature seed thickness (mm) | 150 | 25.0 |
| 100 immature seed weight (g) | 149 | 25.5 |
| **Qualitative traits:** |  |  |
| Seed shape | 199 | 0.5 |
| Seed coat color | 189 | 5.5 |
| Hilum color | 177 | 11.5 |
| Hypocotyl coloration | 199 | 0.5 |
| Stem color | 182 | 9.0 |
| Number of branches | 199 | 0.5 |
| Leaflet size | 150 | 25.0 |
| Leaflet shape | 147 | 26.5 |
| Leaf color | 150 | 25.0 |
| Plant type | 149 | 25.5 |
| Pubescence density | 199 | 0.5 |
| Pubescence color | 197 | 1.5 |
| Corolla color | 199 | 0.5 |
| Pod set capacity | 149 | 25.5 |
| Abbreviation: N, number of accessions.  ^a^Missing rates were calculated based on 200 accessions. | | |

**Supplementary Table 4.** Summary results of imputed phenotypic traits by chained equation in multiple imputation

| Trait | 1^st^ stage multiple imputation | | |  | 2^nd^ stage multiple imputation^a^ | | |
| --- | --- | --- | --- | --- | --- | --- | --- |
|  | $\hat{R}$ of mean | $\hat{R}$ of SD | Convergent |  | $\hat{R}$ of mean | $\hat{R}$ of SD | Convergent |
| **Quantitative traits:** |  |  |  |  |  |  |  |
| Seed length (mm) |  |  |  |  |  |  |  |
| Seed width (mm) |  |  |  |  |  |  |  |
| Seed thickness (mm) |  |  |  |  |  |  |  |
| 100 seed weight (g) |  |  |  |  |  |  |  |
| Plant height (cm) | 1.162 | 1.340 | X |  | 0.990 | 0.986 | O |
| Leaflet length (cm) |  |  |  |  |  |  |  |
| Leaflet width (cm) |  |  |  |  |  |  |  |
| Pod length (cm) | 1.088 | 1.011 | O |  | 0.989 | 0.986 | O |
| Pod width (cm) | 1.190 | 1.003 | X |  | 0.993 | 0.994 | O |
| Stem length to first pod (cm) | 1.120 | 1.055 | X |  | 0.988 | 0.992 | O |
| Shelling rate (%) | 1.206 | 1.981 | X |  | 0.991 | 1.025 | O |
| Immature seed length (mm) | 1.038 | 0.997 | O |  | 0.986 | 0999 | O |
| Immature seed width (mm) | 1.055 | 3.168 | X |  | 0.992 | 0.997 | O |
| Immature seed thickness (mm) | 1.060 | 1.009 | O |  | 0.987 | 0990 | O |
| 100 immature seed weight (g) | 1.114 | 1.216 | X |  | 0.990 | 0.995 | O |
| **Qualitative traits:** |  |  |  |  |  |  |  |
| Seed shape | 1.000 | 1.000 | O |  | 0.986 | 0.986 | O |
| Seed coat color | 1.430 | 1.084 | X |  | 1.000 | 0.999 | O |
| Hilum color | 1.183 | 1.128 | X |  | 0.986 | 0.987 | O |
| Hypocotyl coloration | 0.998 | 0.998 | O |  | 0.994 | 0.994 | O |
| Stem color | 0.985 | 0.984 | O |  | 0.986 | 0.986 | O |
| Number of branches | 1.109 | 0.994 | X |  | 0.992 | 0.986 | O |
| Leaflet size | 9.867 | 3.621 | X |  | 0.986 | 0.986 | O |
| Leaflet shape | 1.141 | 1.286 | X |  | 0.993 | 0.989 | O |
| Leaf color | 1.212 | 1.234 | X |  | 0.991 | 0.988 | O |
| Plant type | 1.087 | 1.086 | O |  | 1.006 | 0.998 | O |
| Pubescence density | 1.360 | 1.284 | X |  | 0.987 | 0.988 | O |
| Pubescence color | 4.102 | 1.102 | X |  | 1.022 | 0.991 | O |
| Corolla color | 1.000 | 1.000 | O |  | 1.000 | 1.000 | O |
| Pod set capacity | 1.256 | 6.563 | X |  | 0.988 | 0.987 | O |
| Abbreviation: SD, standard deviation; X, not converged; O, converged.  ^a^Missing data were estimated using multiple imputation by chained equations. Each chain is the evolution of an object of missing data frame class with 200 observations on 29 phenotypic traits. The four chains have roughly the same mean for each of completed traits. ^b^The value of $\hat{R}$is a convergence statistic (default is smaller than 1.1). | | | | | | | |

**Supplementary Table 5.** Difference test of phenotypic traits between the core collection (using PowerCore, SPS and SRS methods) and the entire collection in vegetable soybean

| Phenotypic trait | Difference test | | | | | | | |
| --- | --- | --- | --- | --- | --- | --- | --- | --- |
|  | PowerCore | |  | SPS | |  | SRS | |
|  | **p.homo** | **p.diff** |  | **p.homo** | **p.diff** |  | **p.homo** | **p.diff** |
| **Quantitative traits:** | 0.00 | 0.96 |  | 0.69 | 0.21 |  | 0.48 | 0.86 |
| Seed length (mm) | 0.01 | 0.34 |  | 0.89 | 0.22 |  | 0.56 | 0.60 |
| Seed width (mm) | 0.08 | **0.04** |  | 1.00 | 0.27 |  | 0.25 | 0.80 |
| Seed thickness (mm) | 0.00 | 0.23 |  | 0.68 | 0.39 |  | 0.73 | 0.47 |
| 100 seed weight (g) | 0.27 | 0.22 |  | 0.60 | 0.85 |  | 0.88 | 0.79 |
| Plant height (cm) | 0.09 | 0.10 |  | 0.65 | 0.64 |  | 0.12 | 0.10 |
| Leaflet length (cm) | 0.11 | 0.36 |  | 0.27 | 0.66 |  | 0.91 | 0.07 |
| Leaflet width (cm) | 0.13 | 0.11 |  | 0.73 | 0.19 |  | 0.79 | 0.87 |
| Pod length (cm) | 0.12 | 0.19 |  | 0.44 | 0.57 |  | 0.09 | 0.17 |
| Pod width (cm) | 0.48 | 0.67 |  | 0.67 | 0.63 |  | 0.70 | 0.95 |
| Stem length to first pod (cm) | 0.21 | 0.54 |  | 0.48 | 0.90 |  | 0.58 | 0.82 |
| Shelling rate (%) | 0.15 | 0.46 |  | 0.67 | 0.95 |  | 0.69 | 0.71 |
| Immature seed length (mm) | 0.03 | 0.48 |  | 0.91 | 0.84 |  | 0.14 | 0.15 |
| Immature seed width (mm) | 0.30 | 0.48 |  | 0.82 | 0.83 |  | 0.32 | 0.47 |
| Immature seed thickness (mm) | 0.03 | 0.13 |  | 0.41 | 0.75 |  | 0.63 | 0.14 |
| 100 immature seed weight (g) | 0.00 | 0.96 |  | 0.69 | 0.21 |  | 0.48 | 0.86 |
| **Qualitative traits:** |  |  |  |  |  |  |  |  |
| Seed shape |  | 0.51 |  |  | 0.39 |  |  | 0.82 |
| Seed coat color |  | 0.21 |  |  | 0.86 |  |  | 0.24 |
| Hilum color |  | 0.37 |  |  | 0.78 |  |  | 0.75 |
| Hypocotyl coloration |  | 0.44 |  |  | 0.81 |  |  | 0.81 |
| Stem color |  | 0.90 |  |  | 0.96 |  |  | 0.95 |
| Number of branches |  | 0.73 |  |  | 0.40 |  |  | **0.048** |
| Leaflet size |  | 0.68 |  |  | 0.39 |  |  | 0.23 |
| Leaflet shape |  | 0.34 |  |  | 0.92 |  |  | 0.24 |
| Leaf color |  | 0.47 |  |  | 1.00 |  |  | 1.00 |
| Plant type |  | 0.21 |  |  | 1.00 |  |  | 0.60 |
| Pubescence density |  | 0.24 |  |  | 0.91 |  |  | 0.43 |
| Pubescence color |  | 0.34 |  |  | 0.32 |  |  | 0.98 |
| Corolla color |  | 0.35 |  |  | 0.77 |  |  | 0.12 |
| Pod set capacity |  | 0.75 |  |  | 0.59 |  |  | 0.23 |
| Abbreviation: SPS, stratified proportional sampling; SRS, simple random sampling; p.homo, p-value of homogeneity test for variance (Levene’s test); p.diff, p-value of difference test (Student’s *t*-test or Welch’s *t*-test or Chi-squared test).  ^a^Core collection was identified using advanced M strategy (PowerCore), SPS and SRS for mixed-type phenotypic traits, respectively. ^b^Student’s *t*-test (if assumption of homogeneity of variance is met) and Welch’s *t*-test (if assumption of homogeneity of variance is not met) were used to conduct mean difference among two collections for quantitative traits; Chi-squared test was conducted to test for difference among two collections for qualitative traits. | | | | | | | | |

**Supplementary Table 6.** Diversity between the core collection (using PowerCore, SPS and SRS methods) and the entire collection in vegetable soybean

| Phenotypic trait | Diversity | | | | | | | |
| --- | --- | --- | --- | --- | --- | --- | --- | --- |
|  | PowerCore | |  | SPS | |  | SRS | |
|  | H′ | Nei’s |  | H′ | Nei’s |  | H′ | Nei’s |
| **Quantitative traits:** |  |  |  |  |  |  |  |  |
| Seed length (mm) | 0.96 | 0.81 |  | 0.89 | 0.74 |  | 0.93 | 0.79 |
| Seed width (mm) | 0.97 | 0.84 |  | 0.88 | 0.77 |  | 0.93 | 0.76 |
| Seed thickness (mm) | 0.98 | 0.79 |  | 0.92 | 0.75 |  | 0.94 | 0.80 |
| 100 seed weight (g) | 0.92 | 0.85 |  | 0.92 | 0.74 |  | 0.96 | 0.83 |
| Plant height (cm) | 0.95 | 0.86 |  | 0.93 | 0.82 |  | 0.94 | 0.76 |
| Leaflet length (cm) | 0.73 | 0.52 |  | 1.00 | 0.50 |  | 0.72 | 0.50 |
| Leaflet width (cm) | 0.88 | 0.77 |  | 0.93 | 0.82 |  | 0.94 | 0.76 |
| Pod length (cm) | 0.94 | 0.83 |  | 0.83 | 0.75 |  | 0.90 | 0.78 |
| Pod width (cm) | 0.81 | 0.62 |  | 0.91 | 0.70 |  | 0.94 | 0.71 |
| Stem length to first pod (cm) | 0.92 | 0.75 |  | 0.97 | 0.78 |  | 0.92 | 0.83 |
| Shelling rate (%) | 0.85 | 0.66 |  | 0.95 | 0.72 |  | 0.77 | 0.72 |
| Immature seed length (mm) | 0.91 | 0.83 |  | 0.89 | 0.82 |  | 0.98 | 0.79 |
| Immature seed width (mm) | 0.87 | 0.80 |  | 0.82 | 0.69 |  | 0.97 | 0.81 |
| Immature seed thickness (mm) | 0.95 | 0.83 |  | 0.89 | 0.76 |  | 0.97 | 0.82 |
| 100 immature seed weight (g) | 0.92 | 0.78 |  | 0.95 | 0.81 |  | 0.96 | 0.84 |
| **Qualitative traits:** |  |  |  |  |  |  |  |  |
| Seed shape | 0.89 | 0.67 |  | 0.92 | 0.61 |  | 0.81 | 0.62 |
| Seed coat color | 0.77 | 0.66 |  | 0.81 | 0.55 |  | 0.77 | 0.66 |
| Hilum color | 0.82 | 0.64 |  | 0.64 | 0.50 |  | 0.73 | 0.51 |
| Hypocotyl coloration | 0.99 | 0.49 |  | 0.88 | 0.42 |  | 0.88 | 0.42 |
| Stem color | 0.74 | 0.52 |  | 0.72 | 0.50 |  | 0.72 | 0.50 |
| Number of branches | 0.96 | 0.64 |  | 0.99 | 0.66 |  | 0.83 | 0.53 |
| Leaflet size | 0.72 | 0.49 |  | 0.97 | 0.48 |  | 0.99 | 0.49 |
| Leaflet shape | 0.87 | 0.72 |  | 0.94 | 0.71 |  | 0.83 | 0.65 |
| Leaf color | 0.98 | 0.48 |  | 0.90 | 0.43 |  | 0.87 | 0.41 |
| Plant type | 0.59 | 0.24 |  | 0.30 | 0.10 |  | 0 | 0 |
| Pubescence density | 0.91 | 0.75 |  | 0.93 | 0.70 |  | 0.86 | 0.64 |
| Pubescence color | 0.98 | 0.65 |  | 0.95 | 0.62 |  | 0.92 | 0.61 |
| Corolla color | 0.89 | 0.58 |  | 0.65 | 0.39 |  | 0.58 | 0.37 |
| Pod set capacity | 0.90 | 0.60 |  | 0.92 | 0.61 |  | 0.75 | 0.53 |
| Abbreviation: SPS, stratified proportional sampling; SRS, simple random sampling.  ^a^Core collection was identified using advanced M strategy (PowerCore), SPS and SRS for mixed-type phenotypic traits, respectively. | | | | | | | | |

**Supplementary Table 7.** Evaluation of the core collection (using PowerCore, SPS and SRS methods) in percentage of the trait differences with the entire collection in vegetable soybean

| **Method to construct core collection** | **MD%** | **VD%** | **CR%** | **VR%** | **Coverage%^a^** | | |
| --- | --- | --- | --- | --- | --- | --- | --- |
|  |  |  |  |  | **Quantitative traits** | **Qualitative traits** | **Combined traits** |
| **The property of the CC:** |  |  |  |  |  |  |  |
| Advanced M strategy (PowerCore) | 5.72 | 52.16 | 96.56 | 151.68 | NA | 100 | NA |
| Stratified proportional sampling (SPS) | 2.15 | 356.85 | 58.38 | 89.67 | 83.80 | 90.12 | 86.85 |
| Simple random sampling (SRS) | 4.50 | 41.94 | 69.18 | 117.41 | 81.85 | 89.40 | 85.49 |
| **Percentage of significant difference^b^** |  |  |  |  |  |  |  |
| Advanced M strategy (PowerCore) | 3.45 |  |  |  |  |  |  |
| Stratified proportional sampling (SPS) | 0 |  |  |  |  |  |  |
| Simple random sampling (SRS) | 3.45 |  |  |  |  |  |  |
|  |  |  |  |  |  |  |  |
| Abbreviation: MD%, mean difference percentage; VD%, variance difference percentage; CR%, coincidence rate; VR%, variable rate; NA, not available.  ^a^Coverages were computed according to quantitative traits, qualitative traits, and combined traits. ^b^The core collection is considered to be the representative of the entire collection if (1) MD% is small and percentage of significant mean difference (α=0.05) between the core collection and the entire collection in all 29 phenotypic traits is no more than 20%, and (2) the CR% is greater than 80%. | | | | | | | |
